# Conference Report: Functional Magnetic Resonance Imaging for Beginners – A Review of the fMRI Experience IV, 13–14 May 2002, Natcher Conference Center, National Institutes of Health, Bethesda, MD

**DOI:** 10.1100/tsw.2002.878

**Published:** 2002-06-27

**Authors:** Daniel Caggiano, Mateus Joffily

**Affiliations:** The Cognitive Science Laboratory, The Catholic University of America, Washington, DC, USA; Laboratory of Neurobiology, Federal University of Rio de Janeiro, Rio de Janeiro, Brazil

**Keywords:** conference report, fMRI

## Abstract

The fourth fMRI Experience meeting was held at the Bethesda, Maryland campus of the National Institutes of Health on May 13^th^ and 14^th^, 2002. The purpose of the meeting was to provide a platform for students working with functional magnetic resonance imaging (fMRI) to present their research to an international audience of peers. This year’s meeting featured special lectures from Dr. Leslie Ungerleider (“Imaging Mechanisms of Visual Attention”) and Dr. Daniel Weinberger (“Genetic Variation and fMRI Response”).

## INTRODUCTION

Functional Magnetic Resonance Imaging (fMRI) is one of the most recently developed techniques for investigating brain functional processes. Its noninvasive nature and nonreliance on radioactive markers, in addition to its spatial and temporal resolution, make it one of the most promising tools for exploring the active human brain. Many questions surrounding the technique, including which testing paradigms might be best suited for use with fMRI and the relationship between the blood oxygen level dependent (BOLD) signal and the underlying neuronal activity, are still in debate. Despite these concerns, the number of researchers employing fMRI to investigate a wide range of questions in neuroscience grows every year all over the world. This increasing interest has resulted in a large number of novice researchers in the field. For such beginners, an opportunity to address their uncertainties and discuss the pitfalls of the technique with experienced researchers is imperative.

## About the Meeting

Since 1999, the fMRI Experience meeting has provided fMRI beginners an opportunity to present their work and interact with experts in an informal and academic environment. In its first time outside England, this year's conference, organized by Drs. Carl Senior, Cynthia Fu, Tamara Russell, and Michael Beauchamp, was held at the National Institute of Health in the U.S. and hosted over 500 beginning and established researchers. Financial support from the Laboratory of Brain and Cognition of the National Institute of Mental Heath (NIMH) Intramural Research Program, the NIMH Division of Intramural Training and the Institute of Psychiatry in London provided assistance in the form of competitive travel awards for students from 13 different countries, including Australia, Brazil, Canada, China, the Czech Republic, Great Britain, Finland, Hungary, India, Italy, the Netherlands, Turkey, and the U.S.

Each conference day was divided into four sessions – two dedicated to student presentations, and two dedicated to experts in the field to introduce basic aspects of the fMRI technique, data analysis methods, and results from their own work. Two special lectures from Dr. Leslie Ungerleider and Dr. Daniel Weinberger of NIMH were conducted in addition to the oral sessions. Poster sessions provided a further opportunity for students to present their work. All sessions' abstracts are available on-line at http://intramural.nimh.nih.gov/fmriconf/.

## CONFERENCE PROCEEDINGS

Dr. Carl Senior opened the conference by welcoming the participants and addressing the meeting's focus on fMRI beginners. Prof. Steven Williams of the Institute of Psychiatry in London, Dr. Robert Cox of NIMH, and Dr. Peter Bandettini of NIMH introduced the fundamental aspects of fMRI and verified the importance of a good understanding of the underlying processes to assure a proper interpretation of the fMRI data. Williams introduced the physical foundations of the nuclear magnetic resonance and the image generation mechanisms, showing drastic outcomes that may result from ignoring basic safety procedures in the scanner room. Next, Cox presented the problems involved in BOLD signal modeling, mainly focusing on the linear shift-invariant approach to the hemodynamic response. Bandettini complemented these presentations by showing the broad range of variables of which the researcher must be aware when performing an fMRI experiment. The different aspects of the hypothesis- and data-driven approaches to fMRI data analysis were also discussed, introducing a theme that permeated several of the following presentations.

Although early studies of fMRI have focused mainly on hemodynamic responses over somewhat large neural regions, several speakers provided evidence that the data fMRI provides may contain much more information than has commonly been recognized. Student presenters Nikolaus Kriegeskorte of the University of Maastricht and David Cox of the Rowland Institute for Science both suggested that patterns of activity from clusters of voxels can be extracted from the fMRI dataset without referring to a priori models of expected hemodynamic response functions. These models do not depend on voxel-wise activation above a statistical threshold to determine whether a particular neural region is associated with a particular behavioral measure. Such multivariate pattern-matching methods of analysis allow for the possibility of testing neural models based on distributed as well as modular patterns of neural activity, which may describe not only what neurons are responsible for a particular phenomenon, but also how they work together in a functional capacity. Dr. James Haxby of NIMH presented findings suggesting the potential utility of such techniques in studying how visual objects are represented in the brain. Haxby suggested that although activation threshold analysis of fMRI data has revealed confined regions of inferior temporal cortex that respond preferentially for faces and landmarks, the pattern of BOLD response in the voxels of inferior temporal cortex, excluding the fusiform face area, can predict accurately whether participants had been viewing faces, with similar findings for other categories of objects. Dr. Alex Martin of NIMH presented results from fMRI experiments supporting the idea that object concepts are represented in the brain by distributed feature-networks and that these networks may provide the foundation for more abstract, object-associated knowledge. Furthermore, Prof. Rainer Goebel of the University of Maastrict introduced the combined use of hypothesis- and data-driven analyses to gather information from an event-related fMRI experiment designed to investigate the cortical mechanisms underlying imagery and spatial analysis in the visual domain. The presentations of Kriegeskorte, Cox, Haxby, Martin, and Goebel all suggest the possible utility of fMRI as a method of testing for distributed processing or population coding within different neural systems, a perspective that contrasts with the modularity assumptions of many functional neuroimaging studies to date.

Several researchers discussed current applications of fMRI to the study of multimodal processing of early sensory information, language, and neural plasticity. Student presenter Mairead MacSweeney of University College of London showed that deaf, but not hearing, native British sign language (BSL) signers activate secondary auditory cortex while viewing grammatical BSL and a-grammatical hand signals (Tic Tac). Both hearing and nonhearing groups activated left posterior temporal-parietal cortex selectively for BSL in the comparison of Tic Tac and BSL conditions, suggesting that the recruitment of auditory cortex in deaf signers for sign language processing may not be specific to linguistic processing. Prof. Helen Neville of the University of Oregon further suggested that visual motion specifically may activate auditory cortex, perhaps even primary auditory cortex, in deaf signers. In addition, Neville noted altered neural circuitry for motion perception in deaf signers in visual as well as auditory cortex, with increased connectivity between V5 and posterior parietal cortex associated with increased peripheral but decreased central motion discrimination capacity in deaf participants. Dr. Guinevere Eden of Georgetown University demonstrated that another clinical population, developmental dyslexics, may also possess altered patterns of activation in the occipito-temporal and posterior parietal cortex in response to motion stimuli. Furthermore, Eden suggested that behavioral interventions designed to improve dyslexics' reading ability may also increase neural responsiveness in some of these regions. Guosheng Ding of Beijing Normal University studied language-processing mechanisms in normal Chinese/English bilinguals and found similar areas of activation in semantic storage systems for both languages but different regions of activation for translating from the participants' first language vs. translating to the participants' first language. Ding suggested that these differences in translating between languages may be due to the subjective difficulty of a second language relative to one's native language. These presentations imply that neural mechanisms subserving the perception of motion as well as early sensory processing areas might possess a particularly high degree of plasticity, and that this plasticity may be related to linguistic experience.

Increasingly, fMRI has been applied to the study of interactions between functional systems, particularly the interaction of emotional and cognitive/perceptual processes. Janaina Mourao Miranda of the Federal University of Rio de Janeiro examined the modulatory role of the amygdala in visual cortex. By comparing this modulatory effect for arousing pictures having a neutral emotional valence with the effect for arousing pictures having an unpleasant emotional valence, Mourao Miranda determined that arousal alone is insufficient to induce coupling of activity in these two regions. Dr. Leslie Ungerleider of NIMH presented data from an fMRI study designed to investigate whether stimulus valence, as measured by the emotion expressed by face stimuli, requires attentional processing resources. In this study, BOLD responses to emotional faces in the amygdala and other structures were greater than responses to neutral faces, but only when the faces were attended, suggesting attention may indeed be required for the processing of stimulus valence. Belinda Liddell of the University of Sydney examined emotional responses to faces in schizophrenics while recording skin conductance responses (SCR). The schizophrenics showed heightened SCR measures and hypoactivity in amygdaloid and prefrontal structures, leading Liddell to conclude that schizophrenics may experience a constant state of fear that is associated with poorer capacity to appraise threatening stimuli and to place sensory experience in an appropriate stimulus context. A process-oriented perspective of these findings, therefore, might suggest that cognitive factors such as attention and decision-making may interact with emotional processing systems at both early and late stages in the assessment of emotional valence.

Several speakers also discussed recently developed applications of fMRI for neuroscience research. Functional neuroimaging of the monkey brain could potentially provide a great deal of insight into the still poorly understood relationship between the BOLD signal and neural activity. Dr. Elizabeth Disbrow of the University of California utilized fMRI parameters similar to those typically used in human studies and found a surprisingly small (approximately 55%) correspondence between areas of BOLD activation in the monkey brain and regions of activity identified through electrophysiological recording. Much of the discordance between the data sets was identified by Disbrow as possibly due to BOLD activation near blood vessels, which suggests that incorporating structural measures of local cerebral vasculature may provide vital information in accurately inferring neuronal activity from hemodynamic changes in fMRI data sets. Dr. Philip McGuire of the Institute of Psychiatry, London discussed ways in which fMRI can be applied to the study of clinical populations such as schizophrenics in order to examine the neural underpinnings of particular clinical symptoms. McGuire's work has demonstrated that although auditory hallucinations may possess some similarities in brain activation patterns with inner speech, these two patterns are not identical. The neuroimaging data, therefore, suggest a more complex neural basis of auditory hallucinations than one might predict from theories that explain auditory hallucinations in schizophrenia as the misidentification of normal inner speech. Transcranial magnetic stimulation (TMS) provides a focal, temporary, reversible disruption of normal neural activity through the application of a brief strong magnetic pulse over the scalp. A useful tool in its own right, TMS can also be combined with structural and functional imaging techniques to provide convergent evidence for the necessity of activity in a neural structure for a particular function. As an example, Dr. Vincent Walsh of the University of Oxford examined the role of previously observed V1 activity in the awareness of visual motion stimuli by manipulating the timing of disruptive pulses to V1, demonstrating that disruption of V1 activity just following stimulation of V5 disturbs the conscious perception of motion. Yet another MRI application discussed by Dr. Derek Jones of the Institute of Psychiatry, London – diffusor tension MRI – offers a great potential for mapping neuronal fiber tracts through the brain. In the second special lecture of the meeting, Dr. Daniel Weinberger of NIMH described his work on genetic influences on behavior and neural response patterns. Weinberger demonstrated that fMRI can reveal strong gene-brain relationships in situations where the gene-behavior relationship is much weaker. Neural efficiency in prefrontal cortex on an N-back working memory task followed a gene-dose pattern, with greater efficiency as the number of copies of the COMT met allele increased. Furthermore, the effect on BOLD activation patterns of administering amphetamines depended on COMT genotype. These researchers demonstrated that the combination of fMRI with other exploratory techniques can provide a better understanding of how information is distributed and processed in the brain as well as the relationship between the BOLD signal and the underlying neuronal activity.

Functional neuroimaging produces large amounts of data every year. This increasing volume argues for the implementation of an efficient management, storage, and exchange of data within the fMRI community. Because new analysis methods are still being developed, one might expect that the application of these methods to data sets from previous experiments might unveil important new information. In an effort to bring these issues to the fore, Dr. Jack Van Horn of Dartmouth College discussed the fMRI Data Center (fMRIDC), which intends to serve as an archival resource of complete raw data sets from peer-reviewed fMRI studies and to make them publicly available for confirmatory and novel analyses by other researches. This kind of initiative benefits all fMRI groups and provides a means of improving the contributions of every fMRI experiment to the brain imaging research literature.

## CONCLUSIONS

The meeting covered a broad range of brain research areas that are benefiting from the neuroimaging approach. Several presentations shared common themes, addressing a set of major applications of the fMRI technique. Many works utilized fMRI data to explore how information is coded and distributed in the brain. The fMRI registration of whole brain activity during behavioral tasks has proven to be an important source of information for the study of multimodal processing and of interactions between functional systems. Furthermore, fMRI is proving to be a valuable tool for studying the normal as well the compromised brain, evidencing its relevance both in clinical and basic research.

The congregation of international scientists to share their experiences and keep apprised of each other’s work is an extremely important part of the scientific process. It is pleasant to know that an fMRI conference initially organized by Ph.D. students and devoted to Ph.D. students is succeeding over the years to bring together an increasing number of novices and experts in the fMRI field.

The quality of the student presentations, ranging from more technical and methodological aspects to the investigation of higher order cognitive phenomena, underscores the relevance of the conference. Keeping in mind that it would be difficult to discuss every fMRI nuance in a 2-day meeting, the conference fulfilled its goals. We hope that in the future the fMRI Experience meeting can be held in different countries in an effort to strengthen fMRI groups all over the world. Next year’s meeting is tentatively scheduled for March 10th and 11th and will be held at Kings College of the University of London, U.K. Interested persons can contact Dr. Tamara Russell at T.Russell@iop.kcl.ac.uk for further information.

## ACKNOWLEDGEMENTS

Both authors contributed equally to the writing of this article.
